# Case Study: Does training of private networks of Family Planning clinicians in urban Pakistan affect service utilization?

**DOI:** 10.1186/1472-698X-10-26

**Published:** 2010-11-09

**Authors:** Asma M Qureshi

**Affiliations:** 1 University Research Company LLC 7200 Wisconsin Avenue, Suite 600 Bethesda, MD 20814 USA

## Abstract

**Background:**

To determine whether training of providers participating in franchise clinic networks is associated with increased Family Planning service use among low-income urban families in Pakistan.

**Methods:**

The study uses 2001 survey data consisting of interviews with 1113 clinical and non-clinical providers working in public and private hospitals/clinics. Data analysis excludes non-clinical providers reducing sample size to 822. Variables for the analysis are divided into client volume, and training in family planning. Regression models are used to compute the association between training and service use in franchise versus private non-franchise clinics.

**Results:**

In franchise clinic networks, staff are 6.5 times more likely to receive family planning training (P = 0.00) relative to private non-franchises. Service use was significantly associated with training (P = 0.00), franchise affiliation (P = 0.01), providers' years of family planning experience (P = 0.02) and the number of trained staff working at government owned clinics (P = 0.00). In this setting, nurses are significantly less likely to receive training compared to doctors (P = 0.00).

**Conclusions:**

These findings suggest that franchises recruit and train various cadres of health workers and training maybe associated with increased service use through improvement in quality of services.

## Background

Pakistan is one of six countries in South Asia that are facing a critical shortage of health workers [1] with an insufficient number of doctors and nurses [2] to meet Millennium Development Goals (MDG) for Health [3] [Table 1].

**Table 1:** Current estimates of health workforce in Pakistan

	2000	2004	2007
Doctors	92,824	113,295	127,859

Nurses	37,528	48,446	62,651

Midwives	22,525	23,559	25,261

LHW	5,443	6,741	9,302

Population/doctor	1473	1316	1225

Population/nurses	3642	3076	2501

Population/midwife	6068	6326	6203

Population/LHW	25113	22108	16845

[3] Sources: Economic Survey of Pakistan 2007-2008 and Pakistan Medical and Dental Council (PMDC) and Pakistan Nursing Council (PNC), Islamabad

There are also inequalities in distribution of health workers with approximately 80,000 doctors in urban areas, and about 36,000 in rural areas. In contrast, there are about 8,000 nurses in urban areas and about 15,000 nurses in rural areas [4]. The majority of physicians comprise male doctors, approximately 75,000 versus 41,000 female physicians [5]. Poor health managerial capacity and inadequate referral mechanisms have led to gross inefficiencies in the public sector; and too often, these referrals are not managed at the basic level because of lack of resources, lack of managerial training, lack of patient-centred care and staff shortages [6].

To overcome these deficiencies in primary health care facilities, contracting out management and delivery of preventive services to NGOs or healthcare providers, has been proposed, underscoring the need for Government to establish a regulatory framework [6].

**Figure 1:**
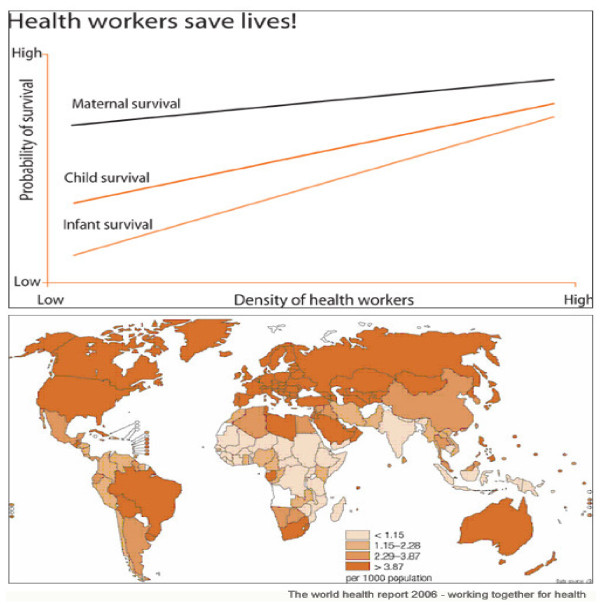
**Density of Health Workers**. Source: http://www.who.int/hrh/hrh_figs_health_workers_large.pdf.

Poor health outcomes are noted for regions with low density of health workers, such as Africa and Southeast Asia as shown below [Figure 1].

More than 50% of maternal deaths in the world are concentrated in six countries and Pakistan is among them [7]. Although progress toward MDG to reduce maternal mortality has been slow in many regions, Tunisia, Malaysia, Jamaica, Sri Lanka and Thailand, have achieved success by investing in health system strengthening, improved access to family planning, emergency obstetric care and skilled birth attendance [8]. Access to family planning services is a complementary strategy to address the burden of maternal mortality because it is estimated that worldwide, meeting unmet need for family planning could reduce maternal mortality by one third [8]. Government of Pakistan's Lady Health Worker program has been successful in improving maternal and infant survival. Lady Health Workers are trained to provide essential drugs, contraceptives, immunizations, antenatal/postnatal services and referrals for safe motherhood [9].

Health system strengthening includes goals for developing an efficient workforce using competency based training methods, mechanisms for supportive supervision, recruitment and deployment of health workers at every level of the health system, and to achieve an appropriate skill mix [10]. Insufficient human resources further require that private sector providers be integrated into public health programs [11-14]. Currently, the private sector in Pakistan provides 70% of health services and the quality of care is perceived to be superior to that of the public sector [6]. In general, utilization of ambulatory care is higher in the private sector and utilization of in-patient care is higher at public sector hospitals because they are better-equipped and the cost of health technology is lower compared to the private sector [6]. Absence of accreditation mechanisms for private hospitals compounds the problem of inadequacies in health delivery [6].

### Social Franchising [15-18]

In developing countries, franchising has emerged as an important strategy for provider training by private sector organizations. A social franchise network is a type of business model, in which a franchising organization, licenses independent providers or service delivery outlets to operate under its brand name. The main difference between social franchising and commercial franchising is that the former focuses on achieving social good such as improved health outcomes for target populations, while the latter is largely for-profit [Table 2].

**Table 2:** Social franchising versus Commercial franchising

	Social franchising	Commercial franchising
Key players	• Franchisor • Franchisee • Donor (pays initial capital)	• Franchisor • Franchisee

Goal	• Sustainability of franchisor and franchisee • Profitability of franchisee • Maximize public health	• Profit for franchisor, franchisee and shareholders

Demand for product/service	• Yes	• Yes

Sector	• Non-profit	• For-profit

Product	• Social services, focus on health	• Consumer goods and services e.g. McDonald

Mass Marketing	• Recognition of brand name • Dissemination of information about access to services	• Recognition of brand name

Payment	• Subsidies	• Market prices

The franchiser bears the financial risk of establishing health services or setting up service delivery outlets. Franchisees contribute equity, usually in the form of their facilities, staff and pre-enrolled clients. Their social capital is an important determinant of their selection for franchise membership and subsequent performance within the network. Social capital refers to their medical background and their reputation. Franchisors can only control franchisees through the business contract. One advantage of social franchising is that services can be scaled up and expanded rapidly.

The quality of care in social franchising is assured by training, evidence-based practice, performance monitoring, supportive supervision, standardization of services and penalties for non-compliance leading to subsequent termination of the contract. The franchisor benefits when franchisees are motivated to continue participating in the network. In general, since franchise affiliation influences providers' perceived benefits for additional clients, expansion of services and revenue generation, they are motivated to maintain the quality of their services and continue to participate. Some providers however, leave the network after receiving training because they do not wish to pay a continuing management fee. In this case, their patients continue to seek care from them because they perceive the quality of the provider and not that of the services. This is due to information asymmetry.

Establishment of a franchise network depends on the availability of a favorable market. In countries where there is professional unemployment, limited FP provision, and a demand for health services, a favorable market exists for development of a successful franchise model. Several programs are operational in Asia, Africa and Latin America with financial support from donors in the US, UK and Germany.

### Context of demand for Family Planning [19,20]

The current total population of Pakistan is about 176 million people with 34% living in urban areas. The health expenditure is 2.4% of GDP and there are 0.7 physicians per 1000 people. Unemployment rate is estimated to be 7.4%. The total fertility rate is high at 4 per woman and contraceptive prevalence rate, all methods, is low at 30%. Unmet need for family planning in urban areas is about 22% and in rural areas is about 26%. Approximately 23% of women 20-24 years old give birth before age 20. Maternal Mortality Rate is estimated to be 276 deaths per 100,000 live births. Among women aged 15-49 years, maternal deaths account for about 20% of all female deaths.

### Green Star Network/Social Marketing Pakistan [21,22]

Under the sponsorship of Population Services International, Social Marketing Pakistan (SMP), a non-profit organization, launched the Green Star Franchise Network in 1991. Since its inception, Green Star has trained over 17,000 providers (male and female physicians, female paramedics, pharmacists) in 40 cities across Pakistan. Following the establishment of Green Star, The Futures Group launched the Key Social Marketing network. Providers have identified free training and increased clientele as important incentives for joining the network. Currently, only the Population Welfare Program of the Government of Pakistan provides more family planning services and products than Green Star. General practitioners including male and female physicians and lady health workers, who own private clinics, are invited to join the network through the Pakistan Medical Association. Their clinics must serve a minimum number of patients and have access to ambulances, labs, pharmacy and radiological services. Training of female doctors and lady health workers consists of education about modern contraceptive technology, management of side effects, counseling skills and infection control. The course includes supervised IUCD practice. In contrast, male physicians and nurses receive a short course, which excludes IUCD practice. After completion of training, a contract allows providers to operate under the brand name of Green Star with supplies and equipment necessary for family planning practice. Trainers are generally required to have a medical degree and one year training in obstetrics and gynecology. They also provide follow-up to reinforce knowledge/skills, record keeping, facilitate implementation of infection control and provide family planning products.

This paper investigates the association between training in franchise networks and use of Family planning services among low-income urban families in Pakistan. A related objective is to describe factors that influence training status in order to improve service use at franchise clinics. Government and non-governmental organizations (NGO) are included in the analysis.

## Methods

The data for this study comes from provider interview surveys conducted by A.C. Nielsen, Pakistan in 2001. Data collection was based on multi-stage, multi-cluster surveys of health facilities, clients and providers. Separate questionnaires were used for health facilities, clients and providers working at these facilities. 11 cities with 3 population groups were selected for the survey using stratified random sampling with differential probability of selection. In the first stage of sampling, all health establishments within the cities were listed and low-income areas were sampled using Probability Proportional to Size (PPS). 993 health facilities were systematically selected from areas where franchises were located. The actual sample included government reproductive health centers, non-governmental organizations, private health facilities and pharmacies. Private sector facilities were further divided into franchise and non-franchise. All franchises in each selected city were sampled [23].

In the second stage, the total number of health staff was recorded and authorized family planning providers in all health establishments were interviewed in-person. The actual sample consisted of 1,113 selected providers including doctors, nurses, lady health workers, paramedics and pharmacists/others. Baseline survey data was collected between January and September 2001 and a follow-up survey was completed in August 2004. For this analysis baseline provider interview data was used.

### Survey instrument

The survey was based on a standard 142-item questionnaire, which was broadly divided into measures of service volume, training experience, source and content of training, and referral mechanisms in family planning and post-abortion care respectively. Each health establishment was coded on the questionnaire and the respondent's gender was recorded [Additional file 1].

### Ethics

Informed consent was obtained from all participants. The study was approved by The University of North Carolina School of Public Health Institutional Review Board.

### Measurement

For this analysis, variables of interest related to training intervention and provider outcomes are shown in Table 3.

**Table 3:** Measurement of Variables

*Outcome variables*	*Operationalized by:*
	

Total client volume	"How many clients received Family Planning services from you last week?"

Training in Family Planning care	"Have you ever received in-service training in Family Planning care?"

Source of training	"Who provided this training?"

Training in Post-Abortion Care	"Have you ever received in-service training in post abortion care?"

Source of training	"Who provided this training?"

	

*Independent variables*	

Years of experience providing general healthcare	"How many years of experience providing healthcare do you have in total?"

Years of experience providing family planning care	"How many years of experience providing Family Planning care do you have in total?"

Ever worked for government	"Have you ever worked for the government as a health provider?"

Affiliation with Green Star Network	"Are you affiliated with the Green Star Network?"

Affiliation with Key Social Marketing	"Are you affiliated with the Key Network?"

Time spent on FP services	"How many hours on average per week do you spend providing family planning services?"

*Levels of staff, gender of staff and type of health establishment are coded on the survey

Linear regression models were used to measure the association of training with client volume. Log of total client volume was used to model this effect and the variable of interest was ever received training.

Logistic regression models were used to assess the odds of training in family planning. Type of health establishment was the key variable for determining whether training was received through participation in franchise networks. The models controlled for providers' years of work experience in general healthcare or family planning, gender, staff level, commitment to family planning provision and affiliation with franchise. STATA statistical software was used throughout. In this study pharmacists and pharmacies were excluded from the analysis, reducing the total sample size to 822.

## Results

Table 4 shows the distribution of the total sample of 822 providers in the survey. The mean of the total client volume was about ten. 70% of the providers were trained in Family Planning and approximately 20% had received training in Post-Abortion Care. Among them, 29% received Family Planning training from Government of Pakistan, and 41% were trained in the private sector by franchises and NGOs. Regarding Post-Abortion Care training, 13% were trained by Government, 7% were trained by franchises and NGOs and 1% reported other source of training. 68% of providers worked in the private sector and 32% worked in the public sector. Female doctors and lady health workers comprised the majority of the sample compared to only 7% of nurses and 33% of male doctors. More than half of all providers reported less than 10 years of general healthcare experience and approximately 70% had less than 10 years of Family Planning experience. About 20% of providers devoted 40 hours per week to Family Planning practice. 62% of providers had ever worked for Government while 64% were affiliated with a franchise.

**Table 4:** Distribution of all clinical providers (N = 822)

	*%*	*n*
**Mean of total client volume = 10**		
		
**Ever received training in Family Planning**		
Yes	70	574
No	30	248
		
**Source of training**		
No training	30	248
Green Star	30	248
Key	7	55
NGO	4	37
Government	29	234
		
**Ever received training in Post Abortion Care**		
Yes	21	171
No	79	651
		
**Source of training**		
No training	79	651
Green Star	6	47
NGO	1	12
Govt.	13	108
Other	1	4
		
**Type of health establishment**		
Private non-franchise	18	146
Franchise	46	382
Govt.	4	262
NGO	32	32
		
**Provider designation**		
Doctor	56	456
Nurse	7	58
Lady health worker	37	308
		
**Provider gender**		
Female	67	554
Male	33	268
		
**Duration of general healthcare experience**		
Less than 10 years	55	456
More than 10 years	45	366
		
**Duration of Family Planning experience**		
Less than 10 years	69	564
More than 10 years	31	258
		
**Average hrs/wk spent on Family Planning**		
Less than 20 hrs	57	470
21-40 hrs	23	186
More than 40 hrs	20	166
		
**Ever worked for govt**.		
Yes	62	509
No	38	313
		
**Affiliation with Green Star**		
Yes	45	369
No	55	453
		
**Affiliation with Key**		
Yes	19	153
No	81	669

Does not include missing values

### Training

Table 5 shows the distribution of trained providers in the sample. Across all types of health establishments, more than half of all trained staff worked at franchise clinics and about 75% of health workers were affiliated with a franchise. Post-Abortion Care training was provided to more than half of doctors, health workers with less than ten years of experience in either general healthcare or Family Planning and female providers (about 80%). Approximately 70% of providers had less than ten years of family planning experience, suggesting that medical education, gender and age were important determinants of selection into franchise networks and subsequent training. 67% of providers reported ever working for government suggesting that this is a key predictor of FP and PAC training received in the public sector. About 30% of trained providers committed 40 hours per week to family planning care.

**Table 5:** Distribution of trained providers (N = 745)

	*Trained in FP*		*Trained in PAC*		*Total*	
	***%***	***n***	***%***	***n***	***%***	***n***

**Type of health establishment**						

Private non-franchise	7	39	8	13	7	52

Franchise	52	296	58	100	53	396

Govt. RHC	38	219	30	51	36	270

NGO	3	20	4	7	4	27

Total		574		171		745

						

**Provider education**						

Doctor	50	290	54	92	51	382

Nurse	4	25	6	11	5	36

LHW	45	259	40	68	44	327

Total		574		171		745

						

**Provider Gender**						

Female	75	430	87	149	78	579

Male	25	144	13	22	22	166

Total		574		171		745

						

**Affiliation with Green Star**						

Yes	50	287	58	99	52	386

No	50	287	42	72	48	359

Total		574		171		745

						

**Affiliation with Key**						

Yes	23	130	25	42	23	172

No	77	444	75	129	77	573

Total		574		171		745

						

**Average hours/week FP work**						

Less than 20 hours	49	282	45	76	48	358

21-40 hours	27	157	33	57	29	214

More than 40 hours	24	135	22	38	23	173

Total		574		171		745

						

**General healthcare experience**						

Less than 10 years	54	307	56	95	54	402

More than 10 years	46	267	44	76	46	343

Total		574		171		745

						

**Family Planning experience**						

Less than 10 years	67	382	67	115	67	497

More than 10 years	33	192	33	56	33	248

Total		574		171		745

						

**Ever worked for Government**						

Yes	67	386	67	115	67	501

No	33	188	33	56	33	244

Total		574		171		745

Table 6 shows that compared to private non-franchises, staff working at franchise clinics and non-government health facilities were significantly more likely to receive training in family planning and post-abortion care (Family Planning: franchise OR = 6.5, P = 0.00; NGO: OR = 4.5, P = 0.00. Post-Abortion Care: franchise OR = 2.0, P = 0.18; NGO: OR = 2.2, P = 0.15). Although working at public facilities significantly increased the likelihood of receiving training in Family Planning (OR = 7.6, P = 0.00) the odds of receiving Post-Abortion Care training were equivocal for staff working at public facilities and private non-franchises. Ever working for government significantly increased the odds of training in Family Planning and Post-Abortion Care (FP: OR = 1.6, P = 0.01. PAC: OR = 1.6, P = 0.02).

**Table 6:** Probability of training in FP and Post Abortion Care (N = 745)

	*FP training*		*PAC training*	
	***OR (CI)***	***P-value***	***OR (CI)***	***P-value***

**Type of health establishment**				

Private non-franchise (reference)				

Franchise	6.5 (2.5, 17)	0.00	2.0 (0.7, 5.1)	0.18

Govt.	7.6 (4.1, 14)	0.00	1.0 (0.5, 2.2)	0.93

NGO	4.5 (1.8, 10.8)	0.00	2.2 (0.8, 6.5)	0.15

				

**Provider education**				

Doctor (reference)				

Nurse	0.2 (0.1, 0.4)	0.00	0.5 (0.3, 1.1)	0.08

Lady Health Worker (LHW)	1.2 (0.7, 2.0)	0.53	0.6 (0.4, 0.9)	0.02

				

**Provider gender**				

Female (reference)				

Male	0.6 (0.4, 0.9)	0.02	0.2 (0.1, 0.3)	0.00

				

**General healthcare experience**				

Less than 10 years (reference)				

More than 10 years	1.3 (0.7, 2.1)	0.39	1.0 (0.6, 1.8)	0.97

				

**Family Planning experience**				

Less than 10 years (reference)				

More than 10 years	1.0 (0.5, 1.7)	0.92	1.1 (0.6, 2.0)	0.79

				

**Ever worked for government**				

No (reference)				

Yes	1.6 (1.1, 2.4)	0.01	1.6 (1.1, 2.5)	0.02

				

**Affiliation with Key Social Marketing**				

No (reference)				

Yes	1.7 (1.0, 2.9)	0.06	1.2 (0.8, 1.9)	0.46

				

**Affiliation with Green Star**				

No (reference)				

Yes	1.2 (0.5, 2.9)	0.65	1.1 (0.5, 2.4)	0.74

Compared to doctors, nurses had significantly lower odds of receiving training in Family Planning and Post-Abortion Care (FP: OR = 0.2, P = 0.00; PAC: OR = 0.5, P = 0.08) and Lady Health Workers had significantly lower odds of receiving training in PAC (OR = 0.6, P = 0.02). Male providers had lower odds of receiving training compared to all female providers (OR = 0.6, P = 0.02)

Years of work experience and affiliation with franchise networks did not influence the odds of training suggesting that training is a benefit of recruitment in the network.

### Use of Family Planning Services

Table 7 shows that there was a significant and positive association between use of services and training (P = 0.00, coeff. = 0.2, SE = 0.0 or 1.2 additional clients per week). Relative to private non-franchises, service use was negatively associated with number of staff working at franchise clinics (P = 0.34, coeff = -0.1, SE = 0.1) and positively associated with the number of trained staff working at public facilities and non-government (NGO) clinics (government: P = 0.00, coeff = 0.3, SE = 0.1 or 1.3 additional clients per week; NGO: P = 0.26, coeff = 0.1, SE = 0.1 or 1.1 additional client per week). Positive associations were also noted for years of family planning experience (P = 0.02, coeff = 0.1, SE = 0.1 or 1.1 additional client per week) and franchise affiliation (Key Social Marketing: P = 0.2, coeff = 0.1, SE = 0.0 or 1.1 additional client per week; Green Star: P = 0.01, coeff = 0.2, SE = 0.1 or 1.2 additional clients per week). Overall, within the private sector, franchise affiliation was associated with 2.3 additional clients per week. Relative to female providers, the number of male providers showed a statistically significant negative association with service use (P = 0.00, coeff = -0.1, SE = 0.0).

**Table 7:** Association between total client volume and training (N = 574)

	*Log Client Volume*	
	***Coefficient (SE)***	***P-value***

**Ever received training**		

No (reference)		

Yes	0.2 (0.0)	0.00

		

**Type of health establishment**		

Private non-franchise (reference)		

Franchise	-0.1 (0.1)	0.34

Govt.	0.3 (0.1)	0.00

NGO	0.1 (0.1)	0.26

		

**General healthcare experience**		

Less than 10 years (reference)		

More than 10 years	0.0 (0.1)	0.43

		

**Family Planning experience**		

Less than 10 years (reference)		

More than 10 years	0.1 (0.1)	0.02

		

**Ever worked for government**		

No (reference)		

Yes	0.0 (0.0)	0.32

		

**Affiliation with Key Social Marketing**		

No (reference)		

Yes	0.1 (0.0)	0.2

		

**Affiliation with Green Star**		

No (reference)		

Yes	0.2 (0.1)	0.01

		

**Provider designation**		

Doctor (reference)		

Nurse	0.0 (0.1)	0.77

Lady Health Worker (LHW)	0.0 (0.1)	0.78

		

**Provider gender**		

Female (reference)		

Male	-0.1(0.0)	0.00

## Discussion

This study revealed some important findings about access to family planning services in urban areas, which may be valid in today's context. Dual practice in Pakistan has continued throughout the years and nurse training has continued to be a significant challenge particularly due to the low prestige associated with the profession and few trained mentors available [24]. Dual practice results from providers' desire to generate more income through private practice due to the prevailing low wages, poor referral mechanisms and lack of resources in the public sector. Often providers who engage in dual practice have the propensity to overprescribe and to make inappropriate use of facilities, staff, health technology and other resources available in the public sector. These providers have patients in public practice, which they divert into their private practice. Information asymmetry may be the underlying cause for this diversion when patients cannot differentiate between the services received in the public sector versus those received in the private sector and remain loyal to the provider. Since the early nineties, franchise programs have complemented Ministry of Health's efforts to improve reproductive health outcomes through training, monitoring and standardization of services [25]. Training increases the use of services and the technical competence of providers. In this way, providers who are trained in the public sector and continue to engage in dual practice are likely to provide better care than those who have not received similar training. Training can also work as a non-financial incentive for them and may improve their attitudes. Additionally in the context of dual practice, social franchising in the private sector links training to quality and access to reproductive health services. Data from 2001 showed that staff working at government owned health facilities were associated with the highest probabilities of training in family planning compared to all private sector health facilities. Not surprisingly, public facilities were associated with higher client volume compared to private non-franchises. The choice of family planning method may influence the pattern of service utilization. A survey in 1999 showed that 60% of women in Karachi who used either pills or condoms obtained them from a pharmacy, and the proportion of women who used long-term contraception such as injectables or implant or intra-uterine devices, was equivalent for either government or private clinics. In contrast, 53% of those surveyed about tubal ligation had undergone the procedure at a private clinic and 47% had used a government clinic [26]. Notably, IUD, injectables and sterilization are the preferred methods for family planning in Pakistan [27].

Data from health establishment surveys conducted in 2001 showed that public facilities in Pakistan, Bihar and Ethiopia were associated with higher family planning client volume compared to private non-franchises [23].

In the private sector, franchises and non-governmental organizations were associated with significantly higher odds of training in family planning and post-abortion care compared to non-franchises. The size of trained staff working at government reproductive health centers and NGO clinics was positively associated with increased client volume. However, the number of trained staff varied across private sector facilities, depending upon service capacity and level of specialization of providers. It is likely that tertiary hospitals in the public sector had higher client volume while, franchise clinics with smaller service capacity compared to private non-franchises, were associated with lower client volume. In order to encourage greater use of services franchise programs expanded services through a larger pool of trained providers and included non-governmental organizations as referral facilities in the network. Therefore, providers who were affiliated with franchise networks and those with more than ten years of family planning experience had higher client volume.

The strongest negative associations were noted for provider gender; and nurses were statistically significantly less likely to receive training in both Family Planning and Post-Abortion Care, compared to doctors. Thus, young female doctors comprised the largest proportion of health workers who were trained in Post-Abortion Care. Nurses are deployed to rural areas hence it is to be expected that very few nurses were interviewed in this sample (7%). Negative judgments about the quality of nursing care may prevent women from consulting nurses in Pakistan, explaining why nurses are underutilized. Given that women often refuse pelvic examination performed by male doctors, in-service training for nurses in family planning and post-abortion care may expand the number of female providers available, particularly for IUDs, which are the method of choice for women seeking birth spacing. Training in combination with information dissemination campaigns would promote the quality of nurses, and enable clients to find value from services. Evaluation of a pay for performance voucher pilot scheme conducted by Green Star in 2008-2009 showed that post-partum family planning use was successful among women who used vouchers for antenatal care and facility-based delivery. In this scheme, vouchers were given to poor women to use for a reproductive health services package and providers were compensated on a fee for performance basis. The pilot was conducted in one district of Punjab with a population of 2.2 million and contraceptive prevalence rate (all methods) of 27 percent. Among post-partum contraception acceptors 26% of women chose long-term methods versus 15% of women who chose injectables, 4% who chose pills, and 2% who chose tubal ligation [28]. Globally, experience has shown that nurses and midwives can provide effective counseling, IUD insertion, family planning and post-abortion care [29-33]. Task shifting of reproductive health services to nurses and midwives in Pakistan, may deserve some attention with careful human resource planning.

It appears that gender gap during the clinical encounter may not be a key concern for some women because male doctors in this survey did have clients. However it is likely that these consultations were for methods other than IUD or for matters other than reproductive health.

Quality of care is negatively influenced by barriers affecting access to services in supply chains, in facilities and equipment, in staff training, in media campaigns, and record keeping [34-43]. In some countries, providers' negative attitudes toward family planning have restricted clients' access to services including Brazil where providers believed that women cannot learn to use barrier methods and Africa where clients' age, marital status, number of children, parity and spousal consent influenced providers' decision to provide contraception [44]. Often, poor working environments adversely affect provider performance by placing them at risk for stress and depression. Increased workloads, difficult interpersonal relations, conflicts in performing professional duties, poor skill mix, health worker shortage, low wages and lack of opportunities for professional development all have a spillover effect on patient outcomes [44]. The Islamabad Declaration on Strengthening Nursing and Midwifery 2007 identified three priority areas that needed attention namely: scaling up midwifery and nursing capacity, developing appropriate skill mix and developing positive workplace environments [45].

Quality of care is of key concern to all stakeholders including health professionals [46,47]. The political, administrative and socioeconomic context strongly influences the quality of family planning programs in developing countries. This study showed that only about one-fifth of the providers surveyed devoted up to 40 hours per week to FP care. This number increased to one-third after training, suggesting that providers' attitudes and counseling skills can be enhanced. Additionally, younger providers may have better attitudes about reproductive health and clients may be more willing to seek services from them [48]. A recent study that used data from 2001 and 2004 surveys of health facilities, providers and clients, measured the quality of services based on the Bruce-Jain Framework, and found greater quality scores for public sector facilities and franchise clinic networks compared to NGOs and private non-franchises [49].

Earlier studies have focused on evaluating the quality, access and cost of reproductive health services provided by clinic franchise networks [23,49]. This study described the likelihood of training for various cadres of providers, and the association between training and quality. In the past decade, unmet need has not changed and health worker shortages have continued. Human resources appear to be skewed in favor of doctors and Lady Health Workers.

There is a severe limitation of this study. Data were based on provider interviews conducted in 2001 and analysis was limited to clinical providers located in urban areas. Their responses may be biased based on attitudes toward dual practice and family planning/post-abortion care.

## Conclusions

Franchise networks monitor and train various cadres of health workers to improve the quality of family planning services in urban areas. Trained providers have technical competencies, counseling skills, have abilities in record keeping and serve more clients. In the private sector, clients would benefit more from services offered by trained providers working at franchise service delivery outlets compared to services offered by untrained providers working in private non-franchise clinics. In 2001, nurses appeared to be underutilized as shown by the small proportion of nurses who were providers of family planning in a nationally representative data and their probability of training compared to doctors was low. In a culture where there are restrictions on health seeking behaviors, it is likely that women would choose to see female providers even those with lesser training than doctors. With in-service training in Family Planning and Post-Abortion Care, nurses and midwives could potentially contribute to expanding accessibility of family planning programs in both private and public sectors.

## Competing interests

The author declares that they have no competing interests.

## Pre-publication history

The pre-publication history for this paper can be accessed here:

http://www.biomedcentral.com/1472-698X/10/26/prepub

## Supplementary Material

Additional file 1 **FP Provider/Staff interview**. Click here for file
