# Supplementary material for: Case Study: Does training of private networks of Family Planning clinicians in urban Pakistan affect service utilization?
Source: BMC Int Health Hum Rights. 2010 Nov 9;10:26. doi: 10.1186/1472-698X-10-26 (PMC2988759; doi:10.1186/1472-698X-10-26)
Supplement: Additional file 1 — FP Provider/Staff interview. [file 1472-698X-10-26-S1.PDF]

Pakistan

(corrected against Urdu)

Alternative Business Models (ABM) for Family Planning Service Delivery Cluster Evaluation 2001

FP Provider/Staff Interview

FINAL
4/28/01

IDENTIFICATION

	Establishment code	
	Serial Number<br>(for office use)<br>(same as est_code)	City Date
	Name and number of ward	
	Income area	Circle one: A B C D
	Interviewer	Code Sig Supervisor
	Back checked by	Coded by
	Entered by	Edited by
	Name and phone number of estab.	
	Address of establishment	
Q-D	Name of staff respondent<br>(See Q502 of HOE survey)	Name:
Q-E	Line number of staff respondent<br>(See Q501 of HOE survey)	

**Interviewer:**	**Q-F**<br>**Interview result:**<br>Completed................1<br>Respondent absent ..2<br>Postponed................3<br>Refused....................4<br>Other (specify)..........5<br>For 3 & 4, specify<br>Time:<br>Day:<br>Reason:	**Interview date:**<br>Day Month Year
**Field Editor's name:**		**No. of visits:**

INTERVIEWER: READ THE FOLLOWING INFORMED CONSENT STATEMENT

"Hello. My name is _________________. I am helping to evaluate health services in this area. This is a project to study how family planning and reproductive health care services are delivered in this country. You may contact Mr. Hassan Farooq of Aftab Associates Ltd. with any questions by phoning (42) 5710987 or by writing to [ADDRESS].

This study is being conducted to understand how health facilities and their staff provide reproductive health services and how clients choose to obtain them. The information collected in this study will help us understand how to improve such services for people living in Pakistan.

Your participation will entail one interview about reproductive health services and will last no longer than 15 minutes. Questions will be asked about this health facility and its staff, and the services you received.

We do not expect there to be any risks associated with your participation in the study. However, you may discuss any concerns you have with the research team.

Participation in this study will not incur any cost to you other than your time.

Every effort will be taken to protect the identity of participants in the study. No subjects will be identified in any report or publication of this study or its results.

You are free to participate in this study. You may choose not to answer any particular questions and you may terminate the interview at any time. We may contact you again in three or four years to follow-up on any developments or changes to reproductive health care and will seek your consent for continued participation in this study then.

This project has been reviewed and approved by the University of North Carolina, School of Public Health Institutional Review Board on Research Involving Human Subjects.

NO.	QUESTION	RESPONSE	GO TO
**Section 0: FAMILY PLANNING SERVICE LOAD**			
Q101	Position code of respondent	Doctor....... 1<br>Nurse....... 2<br>Lady Health Visitor ....3<br>Family welfare worker....... 4<br>Compounder/dispenser ....5<br>Pharmacist ....6<br>Sales staff.......7<br>Other (specify) ....8<br>__________________________	
Q102	**INTERVIEWER: RECORD SEX OF RESPONDENT**	Male .... 1<br>Female 2	
Q103	How many years of experience providing health care do you have in total?	Years	
Q104	How many years of experience providing family planning care do you have in total?	Years	
Q105	Have you ever worked for the government as a health provider?	Yes....... 1<br>No....... 2	➔Q107
Q106	Are you also currently working in a government health center or hospital or as a government community health worker?	Yes....... 1<br>No....... 2	
Q107	Are you affiliated with the Green Star network?	Yes....... 1<br>No....... 2	
Q108	Are you affiliated with the Key network?	Yes....... 1<br>No....... 2	
Q109	Do you work full time in this establishment?	Yes....... 1<br>No....... 2	
Q110	How many hours on average per week do you spend at this service establishment?	Hours _______	
Q111	Of these [HOURS FROM Q110], how many hours on average per week do you spend providing family planning services?	Hours _______<br>MUST BE LESS THAN OR EQUAL TO Q110.	
Q112	How many clients received family planning services from you last week?	Clients _______<br>If none, code 000	

NO.	QUESTION	RESPONSE	GO TO
**CHECK 107: GREEN STAR PROVIDER? YES ↓ NO ➔ Q114**			
Q113	To how many clients did you dispense, administer or prescribe the following methods last week?<br><br>**READ OUT EACH METHOD AND RECORD RESPONSE.**	1- Nova Prescribed _________<br>2- Nova Dispensed _________<br>3- Nova-Ject Prescribed _________<br>4- Nova-Ject Dispensed _________<br>5- Nova-Ject Administered _________<br>6- Sathi Prescribed _________<br>7- Sathi Dispensed _________<br>8- Touch Prescribed _________<br>9- Touch Dispensed _________<br>**FEMALE PROVIDERS ONLY:**<br>10- Multiload Administered _________	
Q114	Do you find you have enough time to provide clients with family planning counseling services?	Yes .... 1<br>No.. 2	
Q115	Which of the following types of records do you complete each time you provide a client with family planning services?<br>**READ OUT.**<br>**CIRCLE ALL THAT APPLY.**	A client record card/form .... 1<br>An entry in the clinic register .... 2<br>Informal notes in a notebook .... 3<br>A payment receipt if a fee is involved .... 4<br>Computer-based record .... 5<br>Other (specify) .... [ ]<br>__________________________<br>None of the above.. 98	
**Section 1: FAMILY PLANNING TRAINING**			
Q116	Have you ever received in-service training in family planning care?	Yes .... 1<br>No.. 2	➔Q124
Q117	When did you last receive in-service family planning training?	Months ago _______	
Q118	Who provided this training?	Green Star .... 1<br>Key.. 2<br>Other NGO .... 3<br>Government... 4<br>Other (specify) .... [ ]	

NO.	QUESTION	RESPONSE	GO TO
Q119	What was the content of the training?<br><br>**CIRCLE ALL MENTIONED**	***Method-Related***<br>A. Male sterilization .... 1<br>B. Female sterilization .... 2<br>C. IUD .... 3<br>D. Oral pills .... 4<br>E. Emergency contraception...................... 5<br>F. Implant .... 6<br>G. Injectable .... 7<br>H. Condom .... 8<br>I. Safe abortion care .... 9<br>J. Post abortion care............................... 10<br>K. STD/HIV .... 11<br>L. Infertility .... 12<br>M. Maternity care .... 13<br>N. Breastfeeding .... 14<br>O. Infant and child health .... 15<br><br>***Service-Related***<br>P. Infection prevention............................ 16<br>Q. FP Counseling .... 17<br>R. Client needs/rights .... 18<br>S. Clinic/outlet management .... 19<br>T. Record keeping .... 20<br>U. Other (specify) .... [ ]	
Q120	How many hours/minutes of travel time from here was the training site?	Minutes _______<br>Don't know 9998	
Q121	How many hours of training did you receive?	Hours _______<br>Don't know.. 998	
Q122	Did you receive any in-service training before this?	Yes.. 1<br>No... 2	 ➔Q124
Q123	Compared with other similar training you have received, was this last training better, the same, or worse in terms of knowledge and skills gained for your FP practice?	Better .... 1<br>Same .... 2<br>Worse .... 3<br>Don't know 8	
**IF RESPONDENT WORKS AT MEDICAL STORE, SKIP ➔Q138**			
**Section 2: POST-ABORTION CARE TRAINING**			
Q124	Do you provide (or help provide) any kind of abortion or post-abortion care services?	Yes.. 1<br>No... 2	
Q125	Have you ever received in-service training for post-abortion care?	Yes.. 1<br>No... 2	 ➔Q131

NO.	QUESTION	RESPONSE	GO TO
Q126	When did you last receive training for post-abortion care?	Months ago ______	
Q127	Who provided this training?	Green Star .... 1<br>Other NGO .... 2<br>Government.......... 3<br>Other (specify) .... [ ]	
Q128	What was the content of the training?<br><br>**CIRCLE ALL MENTIONED**	***Method-Related***<br>D&C technique .... 1<br>Other technique.......... 2<br><br>***Service-Related***<br>FP counseling.......... 3<br>FP methods .... 4<br>Client needs .... 5<br>Other (specify) .... [ ]	
Q129	How many minutes of travel time from here was the training site?	Minutes ______<br>Don't know 9998	
Q130	How many hours of training did you receive?	Hours ______<br>Don't know.......... 998	
Q131	Did you receive any in-service training before this?	Yes.......... 1<br>No.......... 2	 ➔Q133
Q132	Compared with other similar training you've received, was your last training better, the same, or worse in terms of knowledge and skills gained for your practice?	Better .... 1<br>Same .... 2<br>Worse .... 3<br>Don't know 8	
**Section 3: FAMILY PLANNING REFERRALS**			
Q133	Do you refer clients (elsewhere) for family planning services?	Yes.......... 1<br>No.......... 2	 ➔Q138

NO.	QUESTION	RESPONSE	GO TO
Q134	To where do you refer most of your clients who want family planning services?<br><br>PROBE: Is there one in particular you refer to?	Government hospital .... 1<br>Government health clinic/center .... 2<br>Maternity Home .... 3<br>Private hospital...................................... 4<br>Private clinic .... 5<br>Doctor.. 6<br>Green Star clinic/provider .... 7<br>Green Star Plus clinic/provider .... 8<br>Key clinic/provider .... 9<br>NGO clinic/hospital .... 10<br>Community Health Worker<br>Or LHW .... 11<br>Family welfare worker (FWW)............... 12<br>LHV .... 13<br>Other (specify)....................................... [ ]<br>______________________________________	
Q135	How many hours/minutes of travel time by bus from here is that establishment located?	Hours _______<br>Minutes _______<br>Don't know 9998	
Q136	For how long have you referred family planning clients to this establishment?	Years _______<br>Less than 1 year 00	
Q137	Why do you refer clients to this establishment?<br><br>**CIRCLE ALL THAT APPLY**	Has a reputation for good quality .... 1<br>Has affordable/lower prices........................... 2<br>I have a referral arrangement<br>with establishment .... 3<br>I receive fee for referral .... 4<br>Is the closest establishment available............ 5<br>They offer emergency services...................... 6<br>Other (specify) .... [ ]<br>Other (specify) .... [ ]	
**Section 4: POST-ABORTION CARE REFERRALS**			
Q138	Do you refer clients (elsewhere) for safe abortion/post-abortion care services?	Yes... 1<br>No.. 2	➔END

NO.	QUESTION	RESPONSE	GO TO
Q139	To where do you refer clients who want abortion or post-abortion services?	Government hospital .... 1<br>Government health clinic/center .... 2<br>Maternity Home .... 3<br>Private hospital...................................... 4<br>Private clinic .... 5<br>Doctor.. 6<br>Green Star clinic/provider .... 7<br>Green Star Plus clinic/provider .... 8<br>Key clinic/provider .... 9<br>NGO clinic/hospital .... 10<br>Community Health Worker<br>Or LHW .... 11<br>Family welfare worker........................... 12<br>LHV .... 13<br>Other (specify).. [ ]<br>_______________________________________	
Q140	How many minutes of travel time by bus from here is this establishment located?	Minutes _______<br>Don't know 9998	
Q141	For how long have you referred abortion- or post-abortion-seeking clients to this establishment?	Years _______<br>Less than 1 year 00	
Q142	Why do you refer clients to this establishment?<br><br>**CIRCLE ALL THAT APPLY**	Has a reputation for good quality .... 1<br>Has affordable/lower prices........................... 2<br>I have a referral arrangement<br>with establishment .... 3<br>I receive fee for referral................................ 4<br>Is the closest establishment available............ 5<br>They offer emergency services...................... 6<br>Other (specify) .... [ ]<br>Other (specify) .... [ ]	

INTERVIEWER: THANK RESPONDENT FOR INFORMATION AND MENTION THAT HE OR SHE MAY BE CONTACTED AGAIN AT A FUTURE TIME.
